# Update and prognosis of *Dermacentor* distribution in Germany: Nationwide occurrence of *Dermacentor reticulatus*

**DOI:** 10.3389/fvets.2022.1044597

**Published:** 2022-11-02

**Authors:** Andrea Springer, Alexander Lindau, Julia Probst, Marco Drehmann, Katrin Fachet, Dorothea Thoma, H. Rose Vineer, Madeleine Noll, Gerhard Dobler, Ute Mackenstedt, Christina Strube

**Affiliations:** ^1^Institute for Parasitology, Centre for Infection Medicine, University of Veterinary Medicine, Hanover, Germany; ^2^Department of Parasitology, Institute of Biology, University of Hohenheim, Stuttgart, Germany; ^3^Department of Infection Biology and Microbiomes, Institute of Infection, Veterinary and Ecological Sciences, University of Liverpool, Liverpool, United Kingdom; ^4^Bundeswehr Institute of Microbiology, Munich, Germany

**Keywords:** ticks, *Dermacentor marginatus*, ornate dog tick, meadow tick, ornate sheep tick, range expansion, citizen science

## Abstract

A considerable range expansion of *Dermacentor reticulatus* has been observed in several European countries, which is concerning in the light of its vector function for several pathogens, including *Babesia canis* and tick-borne encephalitis virus (TBEV). The present study provides an update on the distribution of *Dermacentor* ticks in Germany, using a citizen science approach. Ticks were collected by citizens from March 2020 to May 2021, and submitted along with information on the date and location of collection, potential hosts and details about the circumstances of discovery. In total, 3,292 *Dermacentor* specimens were received, of which 76.4% (2,515/3,292) were identified as *D. reticulatus* and 23.0% (758/3,292) as *D. marginatus*, while 0.6% (19/3,292) were too damaged for species-level identification. *Dermacentor reticulatus* was received from all federal states of Germany. Maxent species distribution models predicted suitable environmental conditions for *D. reticulatus* throughout Germany. Findings on the vegetation or on pastured animals without travel history confirmed the occurrence of this tick species as far north as the most northern German federal state Schleswig-Holstein. In contrast, the distribution of *D. marginatus* still appears to be limited to southwestern Germany, although the northward shift of the distribution limit observed in the preceding citizen science study, as compared with previous published distributions, was confirmed. This shift was also predicted by Maxent species distribution models, reflecting the broader distribution of the tick occurrence data contributed by citizens. Most *D. reticulatus* ticks were found on dogs (1,311/1,960, 66.9%), while *D. marginatus* was mainly discovered on hoofed animals (197/621, 31.7%) and humans (182/621, 29.3%). Human tick bites were reported in 0.7% (14/1,960) of host-assigned *D. reticulatus* and 3.4% (21/621) of host-assigned *D. marginatus*. Further studies to investigate an increasing endemisation of *Babesia canis* in Germany as well as the relevance of *D. reticulatus* for TBEV spread throughout the country, e.g., by traveling dogs, are urgently needed. In view of the activity of *D. reticulatus* during winter or the colder months, which complements that of *Ixodes ricinus*, a year-round tick protection of at least dogs is strongly recommended.

## Introduction

Ticks play a pivotal role in public and veterinary health, both as nuisance pests and as vectors of pathogens. Environmental and climatic changes may lead to changes in tick activity, tick occurrence and, consequently, the incidence of tick-borne diseases (1). Therefore, surveillance of tick-borne diseases should include the geographic distribution of ticks (2).

During the last two decades, an increasing number of studies has reported marked habitat expansion of the ornate dog tick, *Dermacentor reticulatus*, in several European countries, including Poland (3, 4), Slovakia (5), the Czech Republic (6), the United Kingdom (7), the Netherlands (8), and Germany (9, 10). This tick species plays an important role in veterinary medicine due to its vector function for *Babesia canis*, the piroplasmid parasite responsible for potentially fatal babesiosis in dogs (11). An increase in canine babesiosis cases has indeed been observed, e.g., in Poland, the United Kingdom and Germany, and linked to the expanded distribution of *D. reticulatus* (3, 12–14). Furthermore, local *D. reticulatus* populations in the Netherlands have recently been incriminated in autochthonous transmission of *Babesia caballi* and *Theileria equi*, the causative agents of equine piroplasmosis (8). Moreover, *D. reticulatus* can transmit zoonotic pathogens such as tick-borne encephalitis virus (TBEV) (15) and spotted-fever group rickettsiae (16).

The underlying causes of the habitat expansion of *D. reticulatus* are probably multi-factorial, involving changes in agricultural practices and land use, such as renaturation of landscapes, increased density and movement of wildlife and domestic animals as well as increased temperatures, which facilitate the completion of the tick's life cycle within 1–2 years (17–20). In contrast to *D. reticulatus*, the range of the only congeneric species in Europe, *Dermacentor marginatus*, seems to be rather stable, although a possible northward expansion along the Rhine has recently been reported (10).

In 2019, a citizen science study was initiated in Germany to collect data on the geographic distribution of *Dermacentor* ticks (10). The study has been continued since and allows an update of the reported distribution, with several new locations with *Dermacentor* infestation risk. In addition, machine learning models reveal the predicted potential distributions of *Dermacentor* spp. throughout Germany based on the citizen data collected and current environmental conditions. The resulting maps can be used to inform public and veterinary health risk assessments (21), which might be especially useful in unsampled, or less well-sampled areas.

## Materials and methods

### Citizen science call

Press releases asking citizens to submit *Dermacentor* ticks, along with information on the date and location of collection (GPS [Global Positioning System] data or postal code), potential hosts and details about the circumstances of discovery, were issued by the University of Veterinary Medicine Hannover, Hanover, northern Germany, as well as the Department of Parasitology at the University of Hohenheim, Stuttgart, southern Germany, during the year 2020. Press releases were circulated in various regional and national media. Additionally, both institutions continuously advocated the citizen science call on their websites, providing pictures to help citizens to distinguish between different tick genera. Apart from this, no additional training was provided.

### Tick identification

Ticks were morphologically identified to species level following Arthur (22), Siuda (23), and Estrada-Peña et al. (24).

Two *D. marginatus* specimens of unusual origin were additionally subjected to PCR and sequencing of the 16S ribosomal RNA gene using primers 16S+1 and 16S−1 (25) to confirm the morphological identification. For this purpose, partial ticks were homogenized in DirectPCR^®^ Lysis Reagent Cell (PEQLAB Biotechnology GmbH, Erlangen, Germany) and incubated with addition of proteinase K at 56°C overnight, followed by 85°C for 45 min. The 25 μl PCR reaction included 1 μl DreamTaq polymerase (Thermo Fisher Scientific, Epsom, UK), 2.5 μl 10x buffer, 1 μl of each primer (10 μM each) and 0.5 μl dNTPs (10 mM each, Roti^®^-Mix PCR 3, Carl Roth, Karlsruhe, Germany). As template, 0.5 μl tick lysate was used. The thermoprofile consisted of initial denaturation at 95°C for 3 min, followed by 38 cycles of 95°C for 30 s, annealing for 30 s and extension at 72°C for 45 s, and final extension at 72°C for 7 min. The annealing temperature was raised (47–48.8°C) during the first seven cycles as described by Mangold et al. (25), and the remaining cycles were performed with an annealing temperature of 50°C. After visualization on 1.5% agarose gels stained with GelRed^®^ (Biotium Inc., Fremont, CA, USA), PCR products were sent for custom Sanger sequencing (Microsynth Seqlab, Göttingen, Germany).

### Classification of reported locations

The accuracy of the reported locations was classified as reported by Drehmann et al. (10). Briefly, (i) a high accuracy was assumed for ticks collected from pastured animals or from the vegetation, (ii) a medium accuracy for unengorged ticks found on dogs or humans during or immediately after a walk, as well as for ticks from cats or wild terrestrial animals and (iii) a low accuracy in cases of engorged ticks found on dogs, ticks found on birds or ticks found in an unsuitable habitat (e.g., inside a house). If no information was provided, or the specimen was detected on dogs or humans traveling large distances, the accuracy was classified as (iv) unknown.

In addition, the status of each tick species on district level was classified based on the number of ticks received per location. All tick findings, irrespective of the accuracy classification, from 2019 to 2021 were considered, including data from Drehmann et al. (10). GPS coordinates were rounded to two decimal digits, i.e., findings with a maximum distance of approximately 1 km were regarded as being from the same location. If only a single tick was received from a location, this was classified as “occurrence.” A number of 2–5 ticks per location was termed “multiple occurrence,” while > 5 ticks, including both male and female specimens, was regarded as “population establishment.” If ticks were received from multiple years and totaled > 5 specimens, including both sexes, this was deemed “endemisation.”

Spatial data were visualized in R v. 4.1.0 (26) with administrative boundaries retrieved *via* the rworldmap package (27) and from the Global Administrative Areas Database (28). Maps include data of the present study, from Drehmann et al. (10) and locations where ticks were collected from the vegetation with the flagging method by the involved research institutions. From the citizen science study, only locations with a high or medium accuracy were pictured as dots.

A second set of maps was generated to visualize confirmed occurrence, i.e., high accuracy findings on the vegetation, on stationary pastured animals or on terrestrial wildlife.

### Modeling potential *Dermacentor* spp. distributions—Data preparation

The potential spatial distributions of *D. reticulatus* and *D. marginatus* were estimated using Maxent v. 3.4.4 (a machine learning method) (29, 30) in R v. 4.1.0 (26). Maxent takes, as input, (1) georeferenced species occurrence records, (2) gridded environmental variables for the region of interest.

Occurrence records, representing locations where *D. reticulatus* (*n* = 682) and *D. marginatus* (*n* = 638) have been recorded in Germany, were obtained from the high accuracy records collected in the present study and the high accuracy records collected by Drehmann et al. (10). To reduce autocorrelation and spatial bias introduced by heterogeneous sampling effort (e.g., potentially more intensive sampling in more populated locations), the occurrence data for each species were further thinned to a distance of 6 km (minimum 6 km between records; corresponding to the resolution of the environmental layers i.e., size of each pixel) using *spThin::thin* (31), resulting in 121 high accuracy occurrence records for *D. reticulatus* and 108 records for *D. marginatus*.

By default, Maxent software selects 10,000 background points at random from the study region. However, since most occurrence datasets suffer from some degree of sample selection bias, it is recommended to select background points with the same bias as the occurrence records (32). For this, the entire high reliability occurrence dataset was thinned to a distance of 6 km, and a continuous “bias” layer generated by normalizing a kernel density estimate [*raster::density* (33)] to values between 0 and 1. This bias layer was then used to weight the selection of 10,000 random points throughout Germany, ensuring that no more than one point was selected per pixel, and that spatial bias in the background point dataset reflected the bias in the occurrence dataset.

Elevation and current bioclimatic (Bioclim) variables were extracted at a 2.5 min resolution from the Worldclim database (34) using the *raster::getData* function (33).

The CORINE Land Cover 2018 dataset v2020_20u1 (CLC2018) was obtained at a 100 m resolution (35). CLC2018 was resampled (nearest neighbor method) and reprojected to match the resolution and projection of the Bioclim datasets using the *raster::projectRaster* function (33). All environmental datasets were then cropped and masked (33) to the boundary of Germany (36). From the Bioclim dataset, a subset of variables were chosen based on their ecological relevance to *Dermacentor* spp. (37, 38). *usdm::vifstep* (39) was used to exclude variables with a variance inflation factor (VIF) exceeding 10, which indicates collinearity (40). Finally, values for the environmental variables corresponding to the locations of the occurrence points (presences) and background points were extracted using *raster::extract* (33) to generate a comma separated values (.csv) file containing the coordinates for each occurrence and background point, a binary variable representing an occurrence (1) or background point (0), and the environmental values associated with each record. Two of the *D. reticulatus*, and one of the *D. marginatus* records could not be matched with environmental data and were removed from further analyses.

### Modeling potential *Dermacentor* spp. distributions—Model development

Maxent models were run and evaluated using *dismo* in R (41). The default Maxent parameters were implemented as they produce robust, well-performing models (42).

Ten replicates were run for each species to evaluate model performance. For each replicate, a random subsample of 70% of the occurrence and background data was used for model training and the remaining 30% used for model testing [*biomod2::SampleMat2* (43)]. The resultant spatial predictions show the relative environmental suitability for each species expressed as a probability (0–1) for each pixel in the area of interest.

Two measures of model accuracy were used to assess each replicate's model output: the area under the receiver operating characteristic curve (AUC) and the true skill statistic (TSS) calculated using the test data. The AUC is a threshold independent measure of model performance, which quantifies the model's ability to predict higher values of suitability in known presence localities compared to background points. This value ranges from 0 to 1, with 0.5 signifying a model's predictive power is no better than random, and values exceeding 0.7 indicating good model performance (44). The TSS balances the sensitivity (true positive rate) and specificity (true negative rate) of a binary model output and was estimated as the maximum sensitivity + specificity – 1. A TSS value exceeding 0 represents a model preforming better than random and a value of 1 indicates perfect model performance (45).

The threshold probability of environmental suitability that maximized model sensitivity and specificity, based on the test data, was also estimated for both species. This threshold can be used to transform the probabilistic output into binary predictions whereby locations with values above this threshold can be interpreted as environmentally suitable for the species, and locations with values below this threshold can be interpreted as largely unsuitable. However, this transformation was not done in this study as it is the authors' opinions that such data reduction obscures subtleties, which may be epidemiologically important for *Dermacentor* spp., such as the potential for species to exist in areas of marginal suitability, and the potential for the public to misinterpret regions below the threshold as “zero risk.”

Environmental variables' contributions to model predictive performance were estimated using the permutation importance, which represents the percentage reduction in model performance (AUC) when each covariate is randomly permuted. Higher permutation importance values indicate a greater contribution of that covariate to model performance. Response plots produced with the Maxent output were inspected for the variables with the highest permutation importance, to describe the change in environmental suitability across the range of the variable(s). For CLC2018, a categorical variable, land cover classifications yielding high probabilities (>0.65) were identified for each replicate to highlight land cover types most often associated with high environmental suitability.

## Results

### Received *Dermacentor* ticks

From March 2020 until the end of May 2021, 3,292 *Dermacentor* ticks collected in Germany were received. Of these, 76.4% (2,515/3,292) were identified as *D. reticulatus* and 23.0% (758/3,292) as *D. marginatus*, while 0.6% (19/3,292) were too damaged for species-level identification. As expected based on the nidicolous lifestyle of larval and nymphal *Dermacentor* ticks, all received ticks were adult specimens and the sex ratio of *D. reticulatus* was 53.6% male (1,349/2,515) vs. 46.3% female (1,165/2,515), while the sex of one *D. reticulatus* specimen (0.04%) was not identifiable due to a deteriorated condition. Regarding *D. marginatus*, 45.8% (347/758) of specimens were male, 54.1% (410/758) female and one specimen (0.1%) was gynandromorph.

### Geographic distribution of received ticks

**Figure 1:**
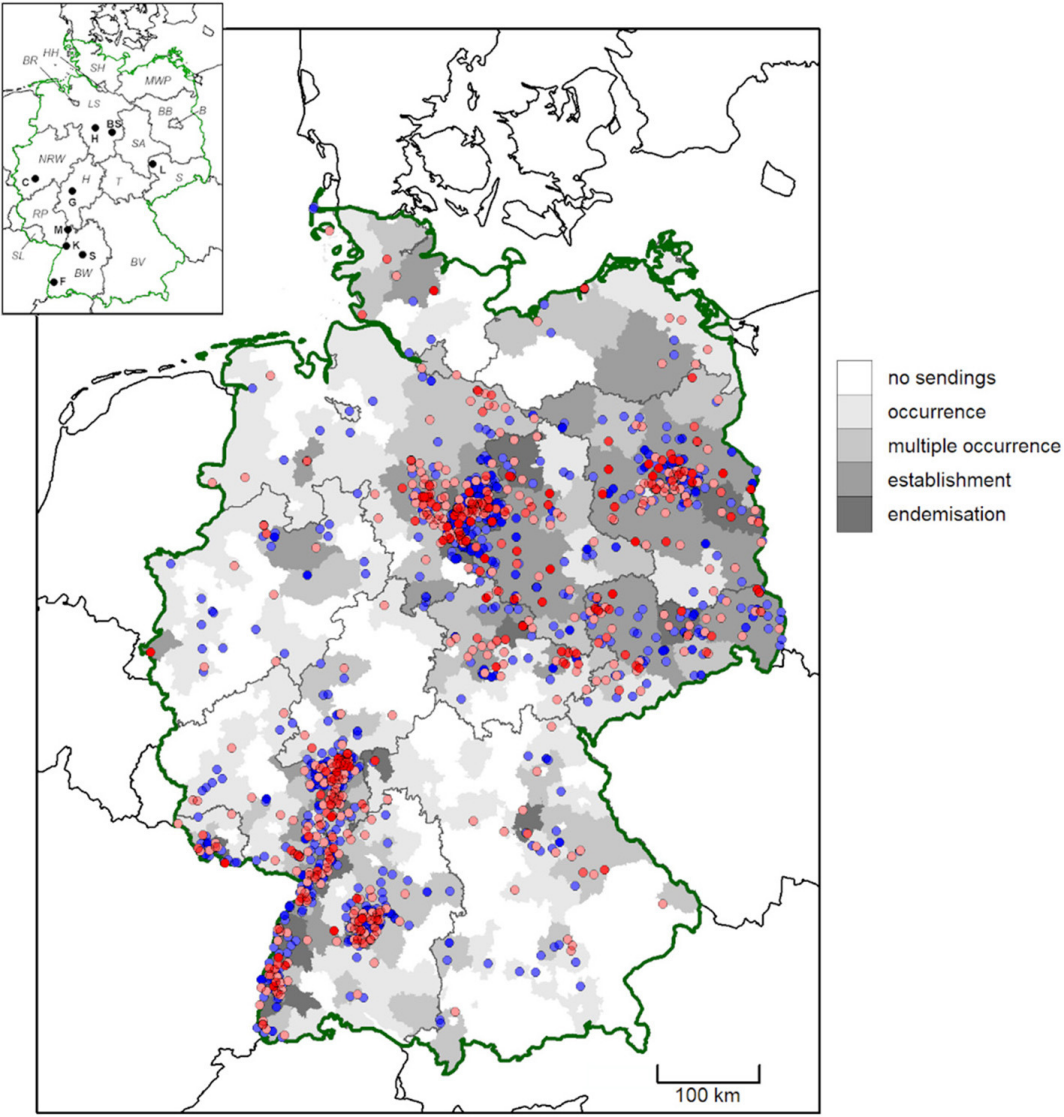
Geographic origin of *D. reticulatus* submissions from German citizens between February 2019 and May 2021. Districts are shaded according to evidence for tick occurrence (single tick/location), multiple occurrence (2–5 ticks/location), establishment (>5 ticks/location) and endemisation (>5 ticks/location, from multiple years). High and medium accuracy records are depicted as dots. Locations previously published by Drehmann et al. (10) are shown in blue, while new records are shown in red. More intense colors indicate multiple findings in close proximity. In the map insert, federal states are abbreviated with italic letters (B, Berlin; BR, Bremen; BW, Baden-Wuerttemberg; BV, Bavaria; BB, Brandenburg; HH, Free and Hanseatic city of Hamburg; H, Hesse; LS, Lower Saxony; MWP, Mecklenburg-Western Pomerania; NRW, North Rhine-Westphalia; RP, Rhineland-Palatinate; S, Saxony; SA, Saxony-Anhalt; SH, Schleswig-Holstein; SL, Saarland; T, Thuringia) and cities with bold letters (BS, Brunswick; C, Cologne; F, Freiburg; G, Gießen; H, Hanover; K, Karlsruhe; L, Leipzig; M, Mannheim; S, Stuttgart).

**Figure 2:**
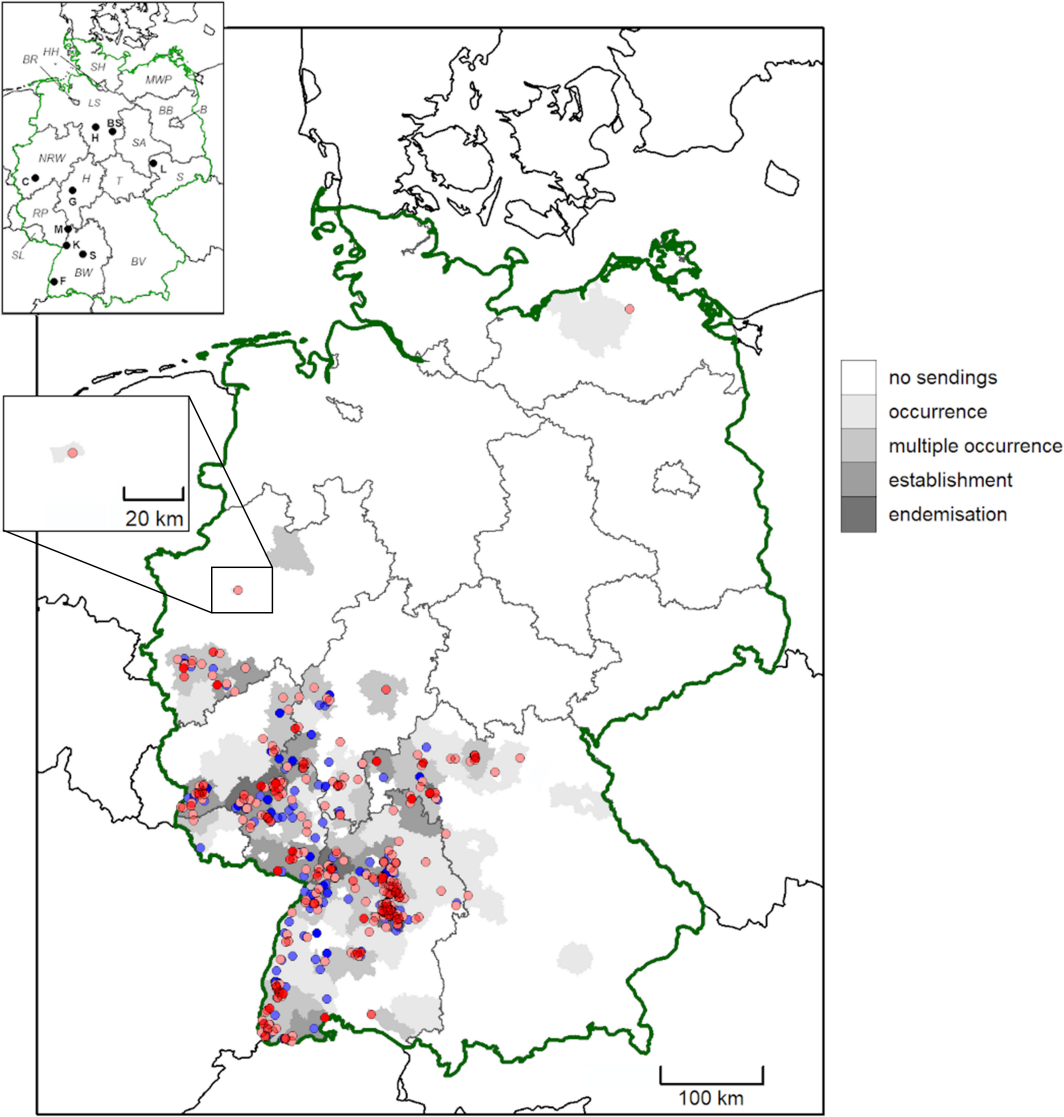
Geographic origin of *D. marginatus* submissions from German citizens between February 2019 and May 2021. Districts are shaded according to evidence for tick occurrence (single tick/location), multiple occurrence (2–5 ticks/location), establishment (>5 ticks/location) and endemisation (>5 ticks/location, from multiple years). High and medium accuracy records are depicted as dots. Locations previously published by Drehmann et al. (10) are shown in blue, while new records are shown in red. More intense colors indicate multiple findings in close proximity. In the map insert, federal states are abbreviated with italic letters (B, Berlin; BR, Bremen; BW, Baden-Wuerttemberg; BV, Bavaria; BB, Brandenburg; HH, Free and Hanseatic city of Hamburg; H, Hesse; LS, Lower Saxony; MWP, Mecklenburg-Western Pomerania; NRW, North Rhine-Westphalia; RP, Rhineland-Palatinate; S, Saxony; SA, Saxony-Anhalt; SH, Schleswig-Holstein; SL, Saarland; T, Thuringia) and cities with bold letters (BS, Brunswick; C, Cologne; F, Freiburg; G, Gießen; H, Hanover; K, Karlsruhe; L, Leipzig; M, Mannheim; S, Stuttgart).

For 98.2% (3,234/3,292) of the ticks, specifically for 97.7% (2,457/2,515) of *D. reticulatus* and 100.0% of *D. marginatus* (758/758), the federal state of origin was unambiguous, whereas the origin was unclear due to missing information or travel activity of the senders for 58 ticks. *Dermacentor reticulatus* was collected in all federal states of Germany, while *D. marginatus* was mainly found in the southwestern part of the country, in the states of Baden-Wuerttemberg, Bavaria, Hesse, North Rhine-Westphalia, Rhineland-Palatinate and Saarland (Table 1, Figures 1, 2). Interestingly, more *D. marginatus* than *D. reticulatus* were sent in from those states, except for Hesse and Saarland (Table 1). Particularly remarkable locations for both tick species are listed in Table 2. A single *D. marginatus* specimen was found on a cat in Mecklenburg-Western Pomerania in northern Germany (Figure 2). This specimen was confirmed as *D. marginatus* by 16S rDNA PCR and sequencing. The obtained sequence showed 99.7% identity to a previously published *D. marginatus* sequence (GenBank acc. no. MH645513, 100% query cover [QC]), while identity to *D. reticulatus* amounted to only 90.1% (acc. no. MT478096, 100% QC). The owners reported that the cat had no travel history. Furthermore, one *D. marginatus* specimen from North Rhine-Westphalia collected in the town of Herne at 7.24° E/51.53° N, i.e., 0.5° north of the currently accepted distribution limit (10, 46), was also confirmed by DNA analysis. The obtained 16S rDNA sequence showed 99.5% identity to publicly available *D. marginatus* sequences (e.g., acc. no. MN907447, 100% QC) and 90.3% identity to *D. reticulatus* (e.g., acc. no. MT478096, 100% QC).

**Table 1:** Distribution of *Dermacentor* ticks with unambiguous origin among the federal states of Germany, received from March 2020 to May 2021.

**Federal state**	***D. reticulatus* (*N* = 2,457)**	***D. marginatus*** **(*N* = 758)**	***Dermacentor* spp. (*N* = 19)**
Baden-Wuerttemberg	283 (11.5%)	403 (53.2%)	4 (21.1%)
Bavaria	45 (1.8%)	83 (10.9%)	0 (0.0%)
Berlin	115 (4.7%)	0 (0.0%)	0 (0.0%)
Brandenburg	399 (16.2%)	0 (0.0%)	1 (5.3%)
Free Hanseatic City of Bremen	1 (0.04%)	0 (0.0%)	0 (0.0%)
Free and Hanseatic City of Hamburg	2 (0.04%)	0 (0.0%)	0 (0.0%)
Hesse	193 (7.9%)	33 (4.4%)	2 (10.5%)
Lower Saxony	507 (20.6%)	0 (0.0%)	3 (15.8%)
Mecklenburg-Western Pomerania	14 (0.6%)	1 (0.1%)	0 (0.0%)
North Rhine-Westphalia	26 (1.1%)	39 (5.1%)	2 (10.5%)
Rhineland-Palatinate	70 (2.8%)	194 (25.6%)	1 (5.3%)
Saarland	47 (1.9%)	5 (0.7%)	0 (0.0%)
Saxony	222 (9.0%)	0 (0.0%)	3 (15.8%)
Saxony-Anhalt	272 (11.1%)	0 (0.0%)	3 (15.8%)
Schleswig-Holstein	58 (2.4%)	0 (0.0%)	0 (0.0%)
Thuringia	203 (8.3%)	0 (0.0%)	0 (0.0%)

**Table 2:** Outstanding locations of *D. reticulatus* and *D. marginatus* findings in Germany.

**Tick species**	**Location**	**District**	**Federal State**	**No. of ticks/engorgement status**	**Host/circumstance of finding**	**Remarks**
*D. reticulatus*	Bordesholm	Rendsburg-Eckernförde	Schleswig-Holstein	50 (22 females, 27 males), mostly engorged	Dog, found after a hunting expedition	
	Dörpstedt	Schleswig-Flensburg	Schleswig-Holstein	2 (1 female, 1 male), unengorged	Horses on pasture	Horses travelled to St. Peter Ording (North Sea coast) 7 days earlier
	Island of Föhr	Nordfriesland	Schleswig-Holstein	1 unengorged female	Dog	
	Island of Rügen	Vorpommern-Rügen	Mecklenburg-Western Pomerania	2 partially engorged males	Dog/inside apartment	2 independent sendings
*D. marginatus*	Behren	Rostock	Mecklenburg-Western Pomerania	1 unengorged female	Cat (no travel history)	Species confirmed by PCR/sequencing
	Herne	Herne	North Rhine-Westphalia	1 unengorged male	Dog	Species confirmed by PCR/sequencing

Taken together, data from Drehmann et al. (10) and the present study period yielded 1,862 individual locations for *D. reticulatus* and 666 for *D. marginatus*. From 1,253 (67.3%) and 511 (76.7%) of these locations, respectively, only single ticks were received, whereas “multiple occurrence” was noted at 467 (25.1%) and 129 (19.4%) locations. From 116 (9.2%) and 20 (3.0%) locations, more than five ticks, including males and females, were received (“establishment”). There was evidence of endemisation, i.e., sendings from multiple years, for 26 (1.4%) locations regarding *D. reticulatus* and 6 (0.9%) locations regarding *D. marginatus*.

In the present study, the accuracy of the reported location of finding was characterized as high for 244 *D. reticulatus* and 289 *D. marginatus* specimens and as medium in 1,206 and 209 cases, respectively. These locations are pictured in Figures 1, 2, together with the locations reported by Drehmann et al. (10) and the evidence for “occurrence,” “multiple occurrence,” “establishment,” and “endemisation” on district level.

### Confirmed occurrence

**Figure 3:**
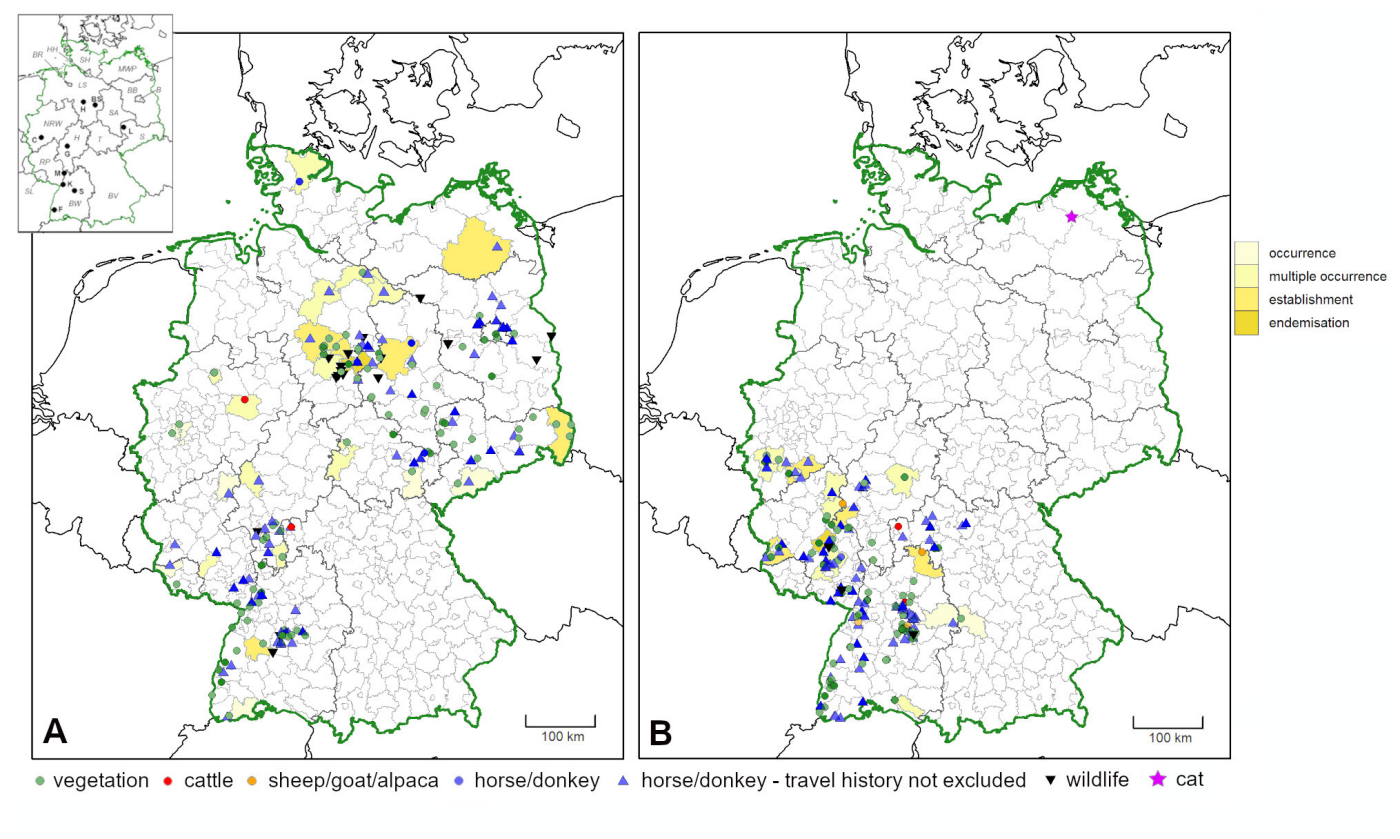
Confirmed occurrence of **(A)** *D. reticulatus* and **(B)** *D. marginatus* in Germany, based on ticks found on the vegetation and infestations on-site or in the closer vicinity, i.e., ticks found on pastured animals and terrestrial wildlife. Additionally, horses/donkeys for which a travel history cannot be entirely excluded were pictured. Maps include data of the present study, from Drehmann et al. (10) and locations where ticks were flagged from the vegetation by the involved research institutions. More intense colors indicate multiple findings in close proximity. Administrative districts with previously unreported confirmed *Dermacentor* occurrence as compared to Rubel et al. (47) are shaded in yellow. In addition, the finding of *D. marginatus* in Mecklenburg-Western Pomerania on a cat without travel history is pictured by a star. In the map insert, federal states are abbreviated with italic letters (B, Berlin; BR, Bremen; BW, Baden-Wuerttemberg; BV, Bavaria; BB, Brandenburg; HH, Free and Hanseatic city of Hamburg; H, Hesse; LS, Lower Saxony; MWP, Mecklenburg-Western Pomerania; NRW, North Rhine-Westphalia; RP, Rhineland-Palatinate; S, Saxony; SA, Saxony-Anhalt; SH, Schleswig-Holstein; SL, Saarland; T, Thuringia) and cities with bold letters (BS, Brunswick; C, Cologne; F, Freiburg; G, Gießen; H, Hanover; K, Karlsruhe; L, Leipzig; M, Mannheim; S, Stuttgart).

Distribution maps of confirmed occurrence for both species based on findings on the vegetation, on stationary pastured animals (on-site infestations) and on terrestrial wildlife (infestations on-site or in the closer vicinity), are shown in Figure 3. As compared to previous findings (47), several additional administrative districts with confirmed *Dermacentor* occurrence, i.e., an infestation/infestation risk within the respective area, were noted (shaded in yellow in Figure 3). However, for most records from horses, a previous travel history cannot be entirely excluded.

Of the 155 individual “high-accuracy” locations for *D. reticulatus*, 77 (49.7%) were based on multiple tick findings. At 15 (9.7%) of these locations, more than five ticks were found, including both male and female specimens, and thus suggesting population establishment. At 10 (6.5%) locations (six in the federal state of Lower Saxony, as well as three in Baden-Wuerttemberg and one location in Thuringia), *D. reticulatus* endemisation was indicated by tick findings in multiple years.

Regarding *D. marginatus*, 152 individual “high-accuracy” locations were recorded, of which 70 (46.1%) were based on multiple tick findings. Population establishment as defined above was suggestive at 16 locations (10.5%), while endemisation was proven by sendings from multiple years in four cases (2.6%).

### Temporal course of citizen's *Dermacentor* collections

**Figure 4:**
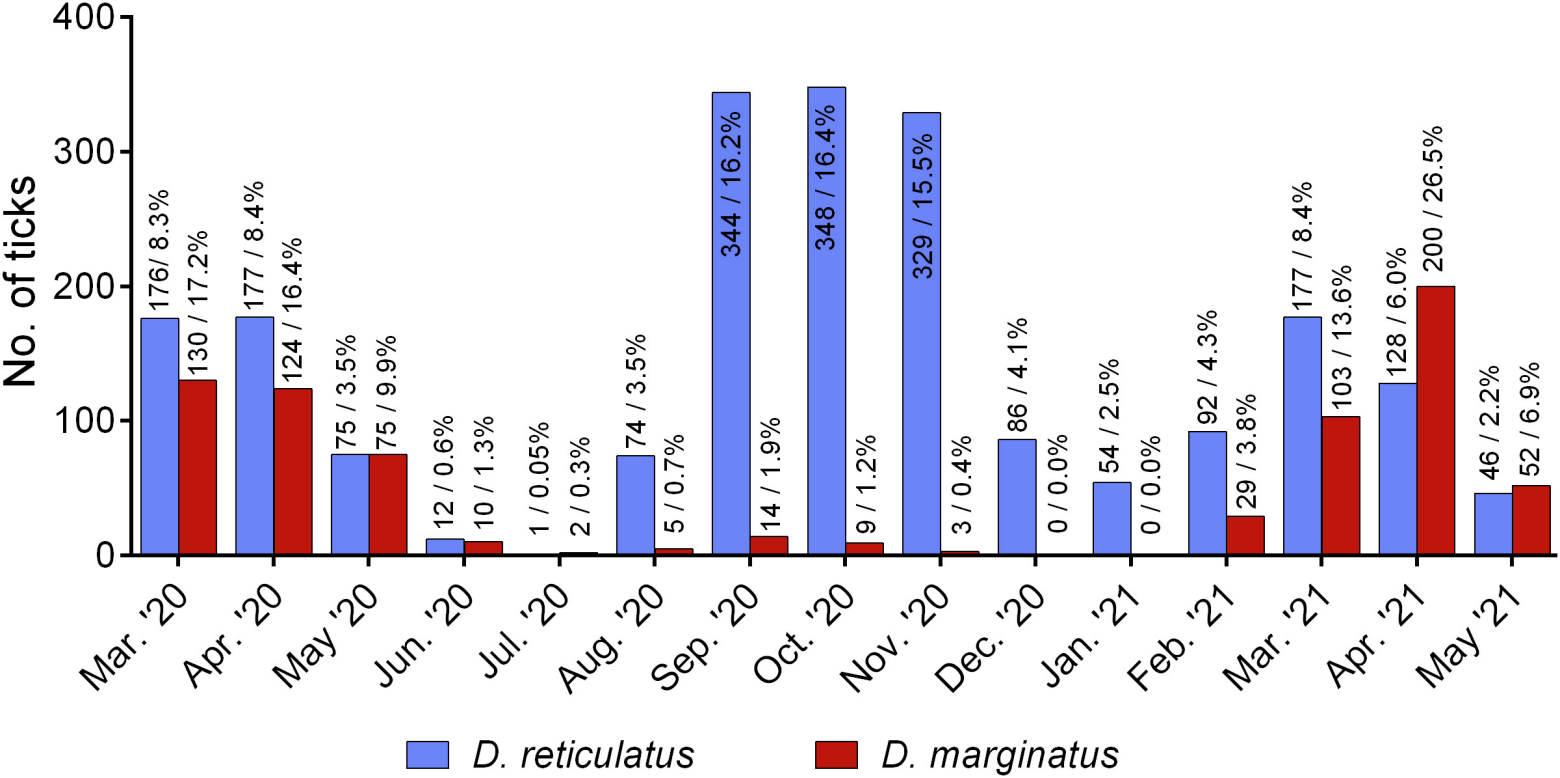
*D. reticulatus* (*N* = 2,119) and *D. marginatus* (*N* = 756) specimens by month of collection, sent in by German citizens from March 2020 to May 2021.

For 2,119/2,515 *D. reticulatus* and 756/758 *D. marginatus* specimens, information on the month of collection was provided. Findings of *D. reticulatus* peaked from September to November 2020, while a smaller peak was noted in March and April of both 2020 and 2021. In contrast, most *D. marginatus* were found in the months of March, April, and May compared to the autumn months (Figure 4).

### Reported host association and human tick bites

Information on host association was available for 1,960/2,515 *D. reticulatus* and 621/758 *D. marginatus* specimens. Detailed information on the host association is presented in Table 3. *Dermacentor reticulatus* was mainly found on domestic animals (1,467/1,960, 74.8%), predominantly dogs (1,311/1,960, 66.9%), while 9.3% (183/1,960) were discovered on the human body and only 22 specimens (1.1%) were associated with wild animals. The remaining 14.7% (288/1,960) were discovered off-host (e.g., on vegetation). *Dermacentor marginatus* was also mainly associated with domestic animals (240/621, 38.6%), especially horses (95/621, 15.3%) and goats (72/621, 11.6%), whereas only a low proportion was found on dogs (41/621, 6.6%). Humans were the putative host in 29.3% of cases (182/621), and 4 specimens (0.6%) were discovered on wildlife. The remaining 31.4% (195/621) were found off-host.

**Table 3:** Host association or location of collection, respectively, for the subset of *Dermacentor* ticks for which this information was available.

**Host/location**	***D. reticulatus* (*N* = 1,960)**	***D. marginatus* (*N* = 621)**	***Dermacentor* spp. (*N* = 15)**
Human	183 (9.3%)	182 (29.3%)	3 (20.0%)
Domestic animals
Dog	1,311 (66.9%)	41 (6.6%)	8 (53.3%)
Cat	7 (0.4%)	2 (0.3%)	0 (0.0%)
Horse	144 (7.3%)	95 (15.3%)	0 (0.0%)
Cattle	4 (0.2%)	28 (4.5%)	0 (0.0%)
Goat	0 (0.0%)	72 (11.6%)	0 (0.0%)
Sheep	0 (0.0%)	2 (0.3%)	0 (0.0%)
Guinea pig	1 (0.1%)	0 (0.0%)	0 (0.0%)
Wildlife
Fox	1 (0.1%)	0 (0.0%)	0 (0.0%)
Cervids	2 (0.1%)	1 (0.2%)	0 (0.0%)
Mouflon	14 (0.7%)	0 (0.0%)	0 (0.0%)
Wild boar	4 (0.2%)	3 (0.5%)	0 (0.0%)
Bird	1 (0.1%)	0 (0.0%)	0 (0.0%)
Off-host
Animal facility (stable/kennel)	2 (0.1%)	0 (0.0%)	0 (0.0%)
Car	3 (0.2%)	7 (1.1%)	0 (0.0%)
Garden	7 (0.4%)	13 (2.1%)	1 (6.7%)
Indoors	165 (8.4%)	78 (12.6%)	3 (20.0%)
Outdoors	102 (5.2%)	87 (14.0%)	0 (0.0%)
Textiles	9 (0.5%)	10 (1.6%)	0 (0.0%)

Human tick bites were reported in 0.7% (14/1,960) of host-assigned *D. reticulatus* (7.7% of all *D. reticulatus* specimens discovered on humans), and 3.4% (21/621) of host-assigned *D. marginatus* (11.5% of specimens discovered on humans).

### Potential *Dermacentor* spp. distributions

**Figure 5:**
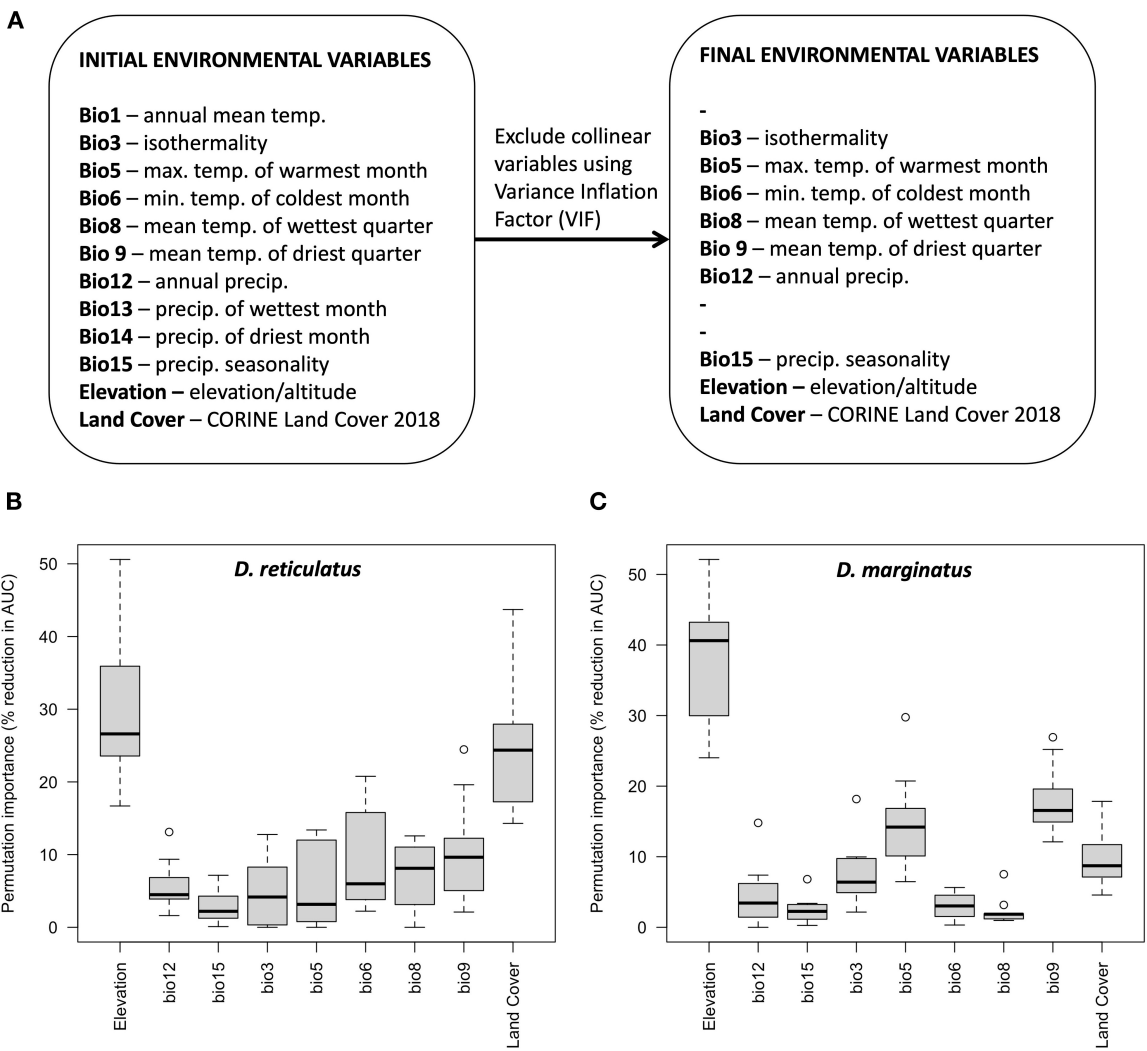
Environmental variable selection for both species initially based on ecological relevance and after stepwise selection to remove collinearity based on the Variance Inflation Factor, VIF **(A)**; and variable permutation importance for *D. reticulatus* **(B)** and *D. marginatus* **(C)**. The permutation importance indicates the percentage reduction in model performance (AUC) when each variable is randomly permuted.

R code and Maxent replicate outputs have been deposited to the Open Science Framework for reuse (48). In total, 119 *D. reticulatus* and 107 *D. marginatus* occurrence points were included in model development. After excluding all environmental variables with VIF > 10, 9 environmental variables remained (Figure 5A). Correlation coefficients for the final selection of environmental variables ranged between −0.76 and 0.04, and VIF ranged between 1.07 and 4.46.

**Figure 6:**
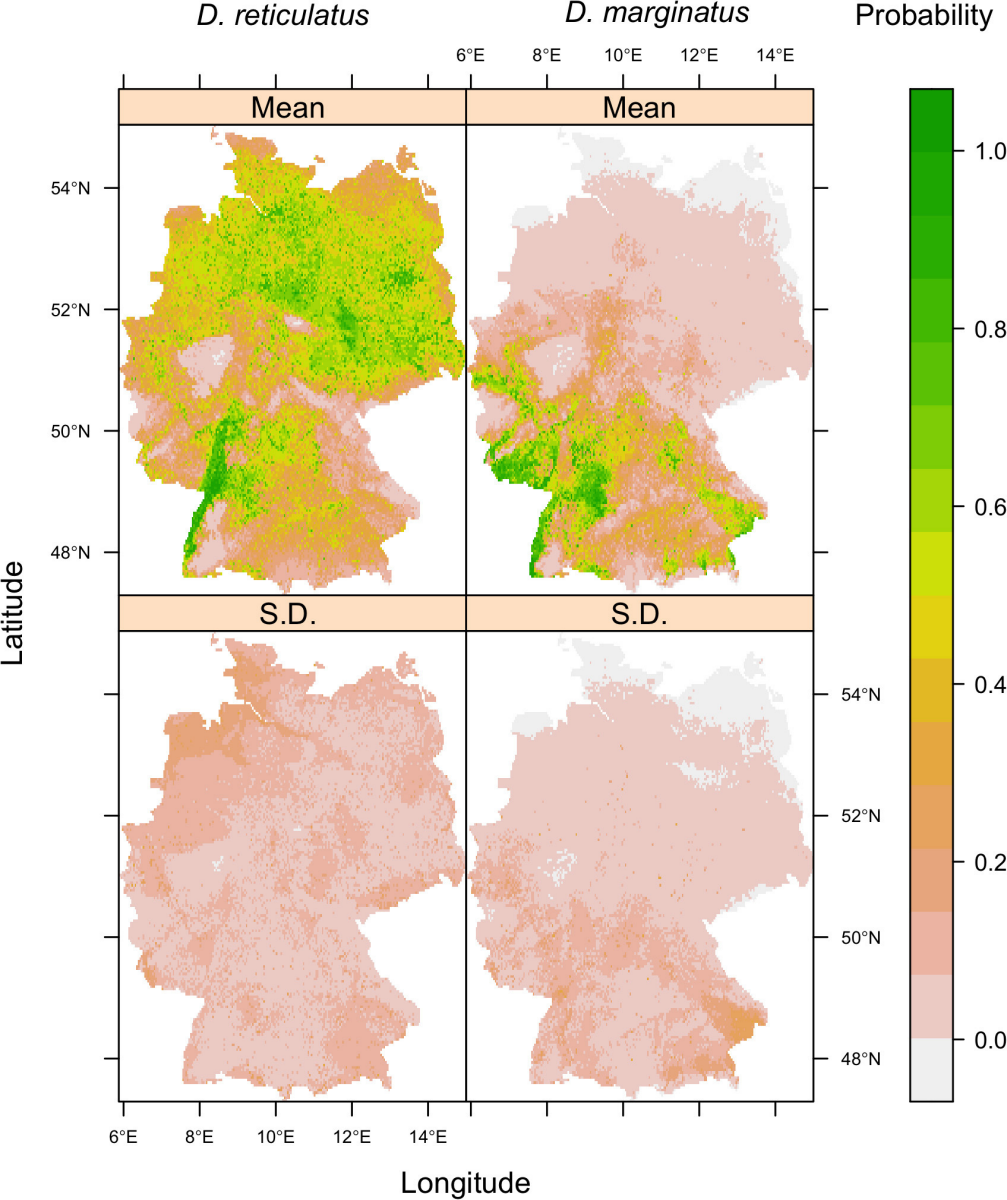
Mean (top row) and standard deviation (bottom row) of 10 Maxent model replicates for *D. reticulatus* (left column) and *D. marginatus* (right column). Model predictions represent the predicted environmental suitability for each species, whereby 0 indicates low potential environmental suitability and 1 indicates a high potential environmental suitability. The threshold probability which maximized sensitivity and specificity was 0.46 (0.11 S.D.) for *D. reticulatus* and 0.24 (0.06) for *D. marginatus*. Each replicate was run using a random 70% subset of the occurrence and pseudoabsence data.

The area predicted by the Maxent models as being environmentally suitable for *D*. reticulatus and *D. marginatus* (Figure 6) encompasses the known geographical distribution of the species (*cf*. Figure 3). The maps highlight certain suitability hotspots, including the Rhine-Plain in the South-West for both species, and the North-East for *D. reticulatus*. The standard deviation for both species' outputs was low across the majority of the study area, indicating consistency between model replicates, with higher deviation in regions with fewer occurrence data points, for example, in the North-West for *D. reticulatus* and South-East for *D. marginatus*. The models for both species performed well statistically (*D. reticulatus* AUC = 0.67 [0.03 S.D.], TSS = 0.31 [0.04]; *D. marginatus* AUC = 0.80 [0.02 S.D.], TSS = 0.56 [0.07]), indicating good predictive capability. The lower AUC values for *D. reticulatus* may be a consequence of the now widespread distribution of this species within Germany, which makes it difficult for the model to discriminate between suitable and unsuitable habitats (49). This is to be expected as it is a known artifact of the AUC. The probability threshold which maximized sensitivity and specificity was 0.46 (0.11 S.D.) for *D. reticulatus* and 0.24 (0.06) for *D. marginatus*.

Among the nine environmental variables, elevation was the most influential and caused the greatest percentage reduction in model performance when permuted for both *D. marginatus* and *D. reticulatus* (Figures 5B,C). When all other variables were held constant, environmental suitability increased with increasing elevation for *D. marginatus*, and decreased with increasing elevation for *D. reticulatus*. Models run using *only* elevation indicated a unimodal relationship with increasing environmental suitability for *D. marginatus* up to ~250 m above sea level, and for *D. reticulatus* up to ~100 m above sea level, decreasing thereafter [see Maxent replicate outputs [response plots and “maxent.html” files]; (48)]. The permutation importance of land cover was also high for *D. reticulatus* (Figure 5B) and a broad range of land classifications were associated with high probabilities of environmental suitability [see Maxent replicate outputs [response plots and “maxent.html” files]; (48)]. This included urban and disturbed land types (“discontinuous urban fabric,” “industrial or commercial units,” “mineral extraction sites,” and “dump sites”), amenity areas (“green urban areas” and “sport and leisure facilities”), fragmented habitats (“discontinuous urban fabric” and “complex cultivation patterns”), “natural grasslands,” and grid cells including “water courses” and “fruit/berry plantations.” Although the permutation importance of land cover for *D. marginatus* was lower, there were interesting differences in the land cover types associated with high probabilities of environmental suitability, which included forest/shrub/natural/fragmented areas (“broad-leaved forest,” “mixed forest,” “traditional woodland-shrub,” “vineyards,” “fruit/berry plantations,” “complex cultivation patterns,” and “land principally occupied by agriculture with significant areas of natural vegetation”) as well as disturbed land (grid cells including “airports” and “mineral extraction sites”). Similarly, for *D. reticulatus cf. D. marginatus*, there was greater variation in importance between the Bioclim variables (Figure 5).

## Discussion

The geographical range expansion of ticks, as observed especially for *D. reticulatus* in Europe (3–10), is concerning in the light of their vector potential. Citizen science studies are useful tools to compile large tick collections with geographic information in a relatively short amount of time, thus helping to assess species distributions across large areas (10, 50, 51). Nevertheless, the data have to be interpreted cautiously, as information provided by citizens can be inaccurate with regard to the origin of the tick, e.g., if a previous travel history is not indicated (51). The present study provides an update of *Dermacentor* distribution in Germany based on a citizen science project initiated in 2019, which classified the accuracy of the obtained records based on the provided information (e.g., travel history), tick engorgement status and mobility of the involved tick host. Consequently, individual locations were only shown in distribution maps when representing “high” and “medium” accuracy records (10), and potential distributions were modeled based on “high” accuracy records only. Nevertheless, finding of a tick at a certain location, even if it did not originate there, indicates transportation of ticks and thus the potential of further range expansion. Therefore, district-level classification of tick occurrence, establishment and endemisation included all obtained records. In addition, “high” accuracy locations, referring to ticks collected from pastured animals, wildlife or directly from the vegetation, were mapped separately to visualize confirmed occurrence of each species in terms of an infestation risk on-site or in the close vicinity.

*Dermacentor* spp. distributions observed and predicted in this study were more widespread than previously reported. As compared to previous findings (47), several additional administrative districts with *Dermacentor* presence were noted. This resulted in a broader range of potential environmental suitability predicted by species distribution modeling, particularly for *D. marginatus*, than previous modeling studies suggest (52), highlighting the importance of ongoing surveillance for accurate risk assessments. Among the nine environmental variables included in the models, elevation was the most influential for both *D. marginatus* and *D. reticulatus*. While environmental suitability increased with increasing elevation for *D. marginatus*, it decreased for *D. reticulatus*. This is consistent with the known distribution of *D. marginatus* at high elevation locations throughout Europe [reviewed by (37)]. Further important variables were land cover for *D. reticulatus* and mean temperature of the driest quarter for *D. marginatus*. For *D. reticulatus*, a greater variation in importance between the Bioclim variables as compared to *D. marginatus* was found, possibly associated with the more generalist life history of *D. reticulatus*. Overall, our bioclimatic model predicted a widespread distribution of environments suitable for *D. reticulatus* in Germany. Predicted probability of environmental suitability was particularly high in established “hotspot” areas (10), but was also elevated throughout much of northern Germany. This reflects the input data, but is also consistent with the finding of range expansion in Germany reported by Drehmann et al. (10).

At approximately half of the locations with confirmed occurrence for each species, multiple ticks were found. Presence of more than five ticks suggested population establishment at ~10% of cases, and findings in multiple years indicated endemisation at 6.5% of high accuracy location for *D. reticulatus* and 2.6% for *D. marginatus*. Additionally, multiple independent “medium” accuracy records in close proximity can also be interpreted as strong evidence of *Dermacentor* endemisation. Based on these data, occurrence of *D. reticulatus* in all federal states of Germany is confirmed, including the northernmost state of Schleswig-Holstein. In this federal state, two *D. reticulatus* specimens, one male and one female, were found on two horses from the same stable. The horse owners confirmed that the horses had no travel history to any other federal state, although they had visited the North Sea coast seven days earlier. However, as both ticks were unengorged it is very unlikely that they were acquired 7 days before they were discovered. Furthermore, several additional *D. reticulatus* specimens were found on dogs in this federal state, e.g., fifty ticks on a single dog after a hunting expedition, and sporadic findings on the tourist destination islands of Sylt and Föhr. This is not surprising, as this tick species has already been detected by flagging the vegetation in climatically similar coastal areas of The Netherlands (53) and at the Baltic coast in the federal state of Mecklenburg-Western Pomerania (54), as well as on a migrating golden jackal in Denmark (55). With regard to the observed clusters of *D. reticulatus* near the cities Hannover and Stuttgart, these are probably due to increased media coverage and citizen participation in proximity to the involved research institutions, as already proposed by Drehmann et al. (10). Nevertheless, *D. reticulatus* endemisation has been proven at several locations near Hanover by flagging ticks from the vegetation in several consecutive years.

In contrast to *D. reticulatus*, a comparable spread of *D. marginatus* is not evident. *D. marginatus* generally has a more southern distribution than *D. reticulatus*, probably due to temperature requirements (52), which is underlined by the fact that *D. marginatus* occurs primarily in traditional vine cultivation regions of Germany. Hence, the land cover type “vineyards” was among the variables associated with high probability of *D. marginatus* occurrence in the species distribution model. However, multiple “high” accuracy findings near Cologne in North-Rhine Westphalia confirm the previously reported expansion to the northwest of the formerly recognized range (10). Occurrence of *D. marginatus* in that area has also been acknowledged in other studies (46). A bioclimatic model, developed using data dating back to the 1970s, suggested a potential *D. marginatus* distribution extending well into northern Germany, with some southern areas of Lower Saxony and Saxony-Anhalt identified as potentially suitable habitat (52). Therefore, the risk of establishment of viable *D. marginatus* populations in that area is given. Our bioclimatic species distribution model, developed using a machine learning method and contemporary records of *Dermacentor* spp. occurrences from this study and Drehmann et al. (10), replicated this, but also predicted a wider distribution of environments suitable for *D. marginatus*, extending into Bavaria and North Rhine-Westphalia. This wider predicted geographic range of environmental suitability included the town of Herne (7.24° E/51.53° N), where a “medium” accuracy record was noted, although 51° N is currently considered the northern distribution limit in Western and Central Europe (10, 46). As only one tick was found on a dog at that location, occurrence of *D. marginatus* in that area needs to be confirmed by further studies. Furthermore, a single *D. marginatus* specimen was found in Mecklenburg-Western Pomerania in the northeast of Germany, at a distance of several hundred kilometers northwards from all other findings. The model did not predict suitable environmental conditions at the location of the record from Mecklenburg-Western Pomerania. However, it is plausible that this tick may have been imported into the area and that this is not representative of an established population. Although the senders reported that the tick was found on a cat without any travel history, the tick may have been transported on another animal or even within a vehicle. For example, another tick sender indicated that he first observed *Dermacentor* ticks within a shipment of hay obtained from a different federal state, before the ticks were repeatedly noticed in the area a year later. This illustrates the danger and routes of tick introduction into previously non-endemic areas.

It should be noted that the bioclimatic species distribution models reported here predict the *potential* range of both *Dermacentor* spp. given the relationships between the environment and the occurrence records used as model input. The *realized* range of a species depends on a number of additional factors, including translocation and host availability, for example. In addition, the predicted distributions may be subject to change when additional data become available—this is evident when comparing the results presented here with the model developed by Walter et al. (52). This is especially the case when modeling species invasions, such as *D. reticulatus*, whereby species distribution models may underpredict the potential range of the species [reviewed by (21)]. However, the species distribution models developed here are valuable tools for targeting future scientific studies, veterinary surveillance, and educating the public in areas of potentially elevated risk. It is recommended that these models are updated periodically to capture potential future changes to these species distributions (56), and as more contemporary data become available.

The temporal course of the citizens' tick collections confirm the bimodal activity pattern of both tick species (38, 57, 58). In contrast to the year 2019, when most of the received *D. reticulatus* specimens were collected in September (10), collection of this species was more evenly spread between the months of September, October and November 2020, which may be due to year-to-year variation of climatic conditions. In 2020, the month of November was particularly warm with an average temperature of 6.0°C, compared to 5.2°C in 2019 (59). However, it should be kept in mind that the temporal course of the collections may have also been influenced by media attention or human behavior. For example, warm temperatures in November 2020 may have resulted in more outdoor activity than in the previous year, and thus increased chances of encountering ticks. In contrast to *D. marginatus, D. reticulatus* was collected throughout the winter months (December to February), confirming winter activity of this tick species. The seasonal pattern of *D. reticulatus* activity in Germany is paralleled by the occurrence of autochthonous canine babesiosis cases, which have become particularly frequent as of 2019 (12, 13), emphasizing the need of year-round tick-protection.

The recorded host association of both species confirms the previously reported pattern, with *D. reticulatus* mainly occurring on dogs and *D. marginatus* mainly on hoofed animals (10). The fact that only few specimens were found on wildlife can be attributed to the citizen science study design, with a relatively low participation of hunters. Interestingly, a larger percentage of both species was found on the human body than in the previous collection period (*D. reticulatus*: 9.34 vs. 4.4%; *D. marginatus*: 29.3 vs. 12.6%) (10). Nevertheless, the proportion of actual tick bites among the specimens encountered on humans remained comparable with the previous report (*D. reticulatus*: 8.2 vs. 7.7%; *D. marginatus*: 13.6 vs. 11.2%) (10). The reason for the increased reports of human *Dermacentor* exposure remain unclear, but it is possible that mostly pet or livestock owners participated in the first year of the study, while continuing media coverage including a highly publicized press release on the range expansion of *Dermacentor* ticks following the publication of Drehmann et al. (10) may have raised attention for the subject of ticks, tick-borne diseases and the citizen science project also among non-animal owners and subsequently increased their participation.

Human exposure to *D. reticulatus* is primarily concerning in light of its vector potential for TBEV. The northern and eastern spread of TBEV in Germany, with more and more districts being declared risk areas (60) as well as further transmission foci detected outside of known risk areas, may thus be linked to the spread of *D. reticulatus* (61, 62). Particularly the recently declared TBEV risk areas in the federal states of Brandenburg and Saxony in eastern Germany (60), near the Polish and Czech borders, were characterized by many *D. reticulatus* records. Expansion of *D. reticulatus* in both of these neighboring countries has been reported (4, 6) and both of them are endemic for TBE. In fact, the Czech Republic has one of the highest incidences in Europe (63). Thus, cross-border transport of ticks, e.g., by migrating wildlife, might drive TBE emergence in eastern Germany.

## Conclusions

In summary, the present data verify the country-wide occurrence of *D. reticulatus* in Germany, including the Free and Hanseatic City of Hamburg, from which no *Dermacentor* ticks had been received previously (10). Furthermore, the northward shift of the distribution limit of *D. marginatus* was confirmed by citizen contributions of tick occurrences, and predictive models of environmental suitability. In addition, predictive modeling suggests a wider distribution of suitable environments than are currently occupied by *Dermacentor* ticks, i.e., a continuing risk of range expansion. Future work should include sampling vegetation to investigate tick endemisation at several locations, especially regarding *D. reticulatus* in the northernmost state of Schleswig-Holstein and regarding the *D. marginatus* findings in the northern part of North-Rhine Westphalia and in Mecklenburg Western-Pomerania. Further studies on increasing endemisation of *Babesia canis*, e.g., by pathogen screening in *Dermacentor* ticks, are highly desirable, as are studies to unravel the role of *D. reticulatus* as a driver of TBEV spread in Germany. Given the currently reported increasing numbers of autochthonous canine babesiosis cases (12, 13) in conjunction with the nationwide *D. reticulatus* occurrence and its activity during winter or the colder months, which complements that of *Ixodes ricinus*, a year-round tick protection of especially dogs all over Germany is strongly recommended. Furthermore, dogs entering Germany should be screened for *B. canis* infection to prevent establishment of further transmission foci due to importation of the pathogen.

## Data availability statement

Maxent model output and R scripts to replicate the models have been deposited to the Open Science Framework for reuse and reanalysis (48). However, due to the nature of this research, participants of this study did not agree for their data to be shared publicly, so supporting tick occurrence data is not available and some metadata associated with the Maxent runs which contain potentially sensitive data (occurrence locations) have been withheld from the repository. Further inquiries can be directed to the corresponding author/s.

## Author contributions

AS: investigation, data curation, formal analysis, visualization, and writing—original draft. AL: investigation and data curation. JP, MD, KF, and DT: investigation. HR: formal analysis, visualization, and writing—review and editing. MN: formal analysis and writing—review and editing. GD: conceptualization and writing—review and editing. UM: conceptualization, project administration, supervision, and writing—review and editing. CS: conceptualization, funding acquisition, project administration, supervision, and writing—review and editing. All authors contributed to the article and approved the submitted version.

## Funding

AS and CS were supported by NorthTick (reference number J-No.: 38-2-7-19), a project co-funded by the European Union through the European Regional Development Fund and the Interreg North Sea Region Programme 2014-2020. This Open Access publication was funded by the Deutsche Forschungsgemeinschaft (DFG, German Research Foundation) - 491094227 Open Access Publication Funding and the University of Veterinary Medicine Hannover, Foundation.

## Conflict of interest

Author CS declares that she repeatedly has lectured for and acted as consultant for (veterinary) pharmaceutical companies and has previous and ongoing research collaborations with various (veterinary) pharmaceutical companies. Authors HR and MN have received funding from, and have an ongoing collaboration with, MSD Animal Health UK (veterinary pharmaceutical company) as part of a separate project to model the impact of climate change on ticks in the UK and Eurasian continent. The remaining authors declare that the research was conducted in the absence of any commercial or financial relationships that could be construed as a potential conflict of interest.

## Publisher's note

All claims expressed in this article are solely those of the authors and do not necessarily represent those of their affiliated organizations, or those of the publisher, the editors and the reviewers. Any product that may be evaluated in this article, or claim that may be made by its manufacturer, is not guaranteed or endorsed by the publisher.

## References

[1] Gray JS Dautel H Estrada-Pena A Kahl O Lindgren E. Effects of climate change on ticks and tick-borne diseases in Europe. Interdiscip Perspect Infect Dis. (2009) 2009:593232. 10.1155/2009/593232 19277106 PMC2648658

[2] Braks M van der Giessen J Kretzschmar M van Pelt W Scholte EJ Reusken C. Towards an integrated approach in surveillance of vector-borne diseases in Europe. Parasit Vectors. (2011) 4:192. 10.1186/1756-3305-4-192 21967706 PMC3199249

[3] Dwuznik-Szarek D Mierzejewska EJ Rodo A Gozdzik K Behnke-Borowczyk J Kiewra D. Monitoring the expansion of *Dermacentor reticulatus* and occurrence of canine babesiosis in Poland in 2016–2018. Parasit Vectors. (2021) 14:267. 10.1186/s13071-021-04758-7 34016152 PMC8138931

[4] Mierzejewska EJ Estrada-Peña A Alsarraf M Kowalec M Bajer A. Mapping of *Dermacentor reticulatus* expansion in Poland in 2012–2014. Ticks Tick Borne Dis. (2016) 7:94–106. 10.1016/j.ttbdis.2015.09.003 26387048

[5] Bullová E Lukán M Stanko M Petko B. Spatial distribution of *Dermacentor reticulatus* tick in Slovakia in the beginning of the 21st century. Vet Parasitol. (2009) 165:357–60. 10.1016/j.vetpar.2009.07.023 19682799

[6] Daněk O Hrazdilová K Kozderková D Jirku D Modrý D. The distribution of *Dermacentor reticulatus* in the Czech Republic re-assessed: citizen science approach to understanding the current distribution of the *Babesia canis* vector. Parasit Vectors. (2022) 15:132. 10.1186/s13071-022-05242-6 35436925 PMC9017003

[7] Medlock JM Hansford KM Vaux AGC Cull B Abdullah S Pietzsch ME. Distribution of the tick *Dermacentor reticulatus* in the United Kingdom. Med Vet Entomol. (2017) 31:281–8. 10.1111/mve.12235 28419493

[8] Jongejan F Ringenier M Putting M Berger L Burgers S Kortekaas R. Novel foci of *Dermacentor reticulatus* ticks infected with *Babesia canis* and *Babesia caballi* in the Netherlands and in Belgium. Parasit Vectors. (2015) 8:232. 10.1186/s13071-015-0841-2 25889392 PMC4404102

[9] Dautel H Dippel C Oehme R Hartelt K Schettler E. Evidence for an increased geographical distribution of *Dermacentor reticulatus* in Germany and detection of *Rickettsia* sp. RpA4. Int J Med Microbiol. (2006) 296:149–56. 10.1016/j.ijmm.2006.01.013 16524777

[10] Drehmann M Springer A Lindau A Fachet K Mai S Thoma D. The spatial distribution of *Dermacentor* ticks (Ixodidae) in Germany - evidence of a continuing spread of *Dermacentor reticulatus*. Front Vet Sci. (2020) 7:578220. 10.3389/fvets.2020.578220 33088837 PMC7544815

[11] Gray JS Estrada-Peña A Zintl A. Vectors of babesiosis. Ann Rev Entomol. (2019) 64:149–65. 10.1146/annurev-ento-011118-111932 30272993

[12] Helm CS Weingart C Ramünke S Schäfer I Müller E von Samson-Himmelstjerna G. High genetic diversity of *Babesia canis* (Piana & Galli-Valerio, 1895) in a recent local outbreak in Berlin/ Brandenburg, Germany. Transbound Emerg Dis. (2022) 69:e3336–45. 10.1111/tbed.14617 35689449

[13] Seibert S Rohrberg A Stockinger A Schaalo S März I. Vorkommen von kaniner Babesiose bei Hunden im Rhein-Main-Gebiet in Hessen – eine Fallstudie mit 81 Hunden. [Occurrence of canine babesiosis in dogs in the Rhine-Main area of Hesse, Germany – a case study of 81 dogs]. Tierarztl Prax Ausg K Kleintiere Heimtiere. (2022) 50:162–72. 10.1055/a-1704-6604 35790164

[14] Wright I. Babesiosis in Essex, UK: monitoring and learning lessons from a novel disease outbreak. Parasit Vectors. (2018) 11:132. 10.1186/s13071-018-2718-7 29554939 PMC5859444

[15] Ličková M Fumačová Havlíková S Sláviková M Slovák M Drexler JF Klempa B. *Dermacentor reticulatus* is a vector of tick-borne encephalitis virus. Ticks Tick Borne Dis. (2020) 11:101414. 10.1016/j.ttbdis.2020.101414 32173297

[16] Földvári G Rigó K Lakos A. Transmission of *Rickettsia slovaca* and *Rickettsia raoultii* by male *Dermacentor marginatus* and *Dermacentor reticulatus* ticks to humans. Diagn Microbiol Infect Dis. (2013) 76:387–9. 10.1016/j.diagmicrobio.2013.03.005 23602788

[17] Földvári G Široký P Szekeres S Majoros G Sprong H. *Dermacentor reticulatus*: a vector on the rise. Parasit Vectors. (2016) 9:314. 10.1186/s13071-016-1599-x 27251148 PMC4888597

[18] Bajer A Rodo A Alsarraf M Dwuznik D Behnke JM Mierzejewska EJ. Abundance of the tick *Dermacentor reticulatus* in an ecosystem of abandoned meadows: experimental intervention and the critical importance of mowing. Vet Parasitol. (2017) 246:70–75. 10.1016/j.vetpar.2017.09.004 28969783

[19] Kloch A Mierzejewska EJ Karbowiak G Slivinska K Alsarraf M Rodo A. Origins of recently emerged foci of the tick *Dermacentor reticulatus* in central Europe inferred from molecular markers. Vet Parasitol. (2017) 237:63–9. 10.1016/j.vetpar.2017.02.020 28285892

[20] Karbowiak G. Changes in the occurrence range of hosts cause the expansion of the ornate dog tick *Dermacentor reticulatus* (Fabricius, 1794) in Poland. Biologia. (2021) 77:1513–22. 10.1007/s11756-021-00945-0

[21] Purse BV Golding N. Tracking the distribution and impacts of diseases with biological records and distribution modelling. Biol J Linn Soc. (2015) 115:664–77. 10.1111/bij.12567

[22] Arthur DR. British Ticks. London: Butterworths (1963).

[23] Siuda K. Kleszcze (Acari: Ixodida) Polski: Cz. 1, Zagadnienia Ogólne. Warsaw: Naukowe PWN (1991).

[24] Estrada-Peña A Mihalca AD Petney TN. Ticks of Europe and North Africa. Cham: Springer International Publishing (2017).

[25] Mangold AJ Bargues MD Mas-Coma S. Mitochondrial 16S rDNA sequences and phylogenetic relationships of species of *Rhipicephalus* and other tick genera among Metastriata (Acari: Ixodidae). Parasitol Res. (1998) 84:478–84. 10.1007/s004360050433 9660138

[26] R Core Team. R: A Language and Environment for Statistical Computing. 4.1.0 ed. Vienna: R Foundation for Statistical Computing (2021).

[27] South A. rworldmap: a New R package for Mapping Global Data. R J. (2011) 3:1:35–43.

[28] GADM. Database of Global Administrative Areas. Available online at: www.gadm.org (accessed June 15, 2020) (2018).

[29] Elith J Phillips SJ Hastie T Dudík M Chee YE Yates CJ. A statistical explanation of MaxEnt for ecologists. Divers Distrib. (2011) 17:43–57. 10.1111/j.1472-4642.2010.00725.x

[30] Phillips SJ Anderson RP Dudík M Schapire RE Blair ME. Opening the black box: an open-source release of Maxent. Ecography. (2017) 40:887–93. 10.1111/ecog.03049

[31] Aiello-Lammens ME Boria RA Radosavljevic A Vilela B Anderson RP. spThin: an R package for spatial thinning of species occurrence records for use in ecological niche models. Ecography. (2015) 38:541–5. 10.1111/ecog.01132

[32] Phillips SJ Dudík M Elith J Graham CH Lehmann A Leathwick J. Sample selection bias and presence-only distribution models: implications for background and pseudo-absence data. Ecol Appl. (2009) 19:181–97. 10.1890/07-2153.1 19323182

[33] Hijmans RJ. raster: Geographic Data Analysis and Modeling. R package version 3.5-15. (2022). Available online at: https://CRAN.R-project.org/package=raster.

[34] Fick SE Hijmans RJ. WorldClim 2: new 1-km spatial resolution climate surfaces for global land areas. Int J Climatol. (2017) 37:4302–15. 10.1002/joc.5086

[35] Copernicus. CORINE Land Cover 2018. Available online at: https://land.copernicus.eu/pan-european/corine-land-cover/clc2018 (accessed June 8, 2022) (2022).

[36] South A. rnaturalearth: World Map Data from Natural Earth. R package version 0.1.0. (2017). Available online at: https://CRAN.R-project.org/package=rnaturalearth.

[37] Rubel F Brugger K Pfeffer M Chitimia-Dobler L Didyk YM Leverenz S. Geographical distribution of *Dermacentor marginatus* and *Dermacentor reticulatus* in Europe. Ticks Tick Borne Dis. (2016) 7:224–33. 10.1016/j.ttbdis.2015.10.015 26552893

[38] Sands BO Bryer KE Wall R. Climate and the seasonal abundance of the tick *Dermacentor reticulatus*. Med Vet Entomol. (2021) 35:434–41. 10.1111/mve.12518 33942903

[39] Naimi B Hamm NAS Groen TA Skidmore AK Toxopeus AG. Where is positional uncertainty a problem for species distribution modelling? Ecography. (2014) 37:191–203. 10.1111/j.1600-0587.2013.00205.x

[40] Guisan A Thuiller W Zimmermann NE. Habitat Suitability and Distribution Models: With Applications in R. Cambridge: Cambridge University Press (2017).

[41] Hijmans RJ Phillips S Leathwick J Elith J. dismo: Species Distribution Modeling. R package version 1.3-5 (2021). Available online at: https://CRAN.R-project.org/package=dismo.

[42] Phillips SJ Dudík M. Modeling of species distributions with Maxent: new extensions and a comprehensive evaluation. Ecography. (2008) 31:161–75. 10.1111/j.0906-7590.2008.5203.x

[43] Thuiller W Georges D Gueguen M Engler R Breiner F. biomod2: Ensemble Platform for Species Distribution Modeling. R package version 3.5.1 (2021). Available online at: https://CRAN.R-project.org/package=biomod2

[44] Elith J Graham CH Anderson RP Dudík M Ferrier S Guisan A. Novel methods improve prediction of species' distributions from occurrence data. Ecography. (2006) 29:129–51. 10.1111/j.2006.0906-7590.04596.x

[45] Allouche O Tsoar A Kadmon R. Assessing the accuracy of species distribution models: prevalence, kappa and the true skill statistic (TSS). J Appl Ecol. (2006) 43:1223–32. 10.1111/j.1365-2664.2006.01214.x

[46] Pluta S. Epidemiologie von Coxiella burnetii, Rickettsia spp., FSME-und Hantaviren in Süddeutschland unter Berücksichtigung klimatischer Veränderungen. [Dissertation]. University of Hohenheim, Hohenheim, Germany (2011).

[47] Rubel F Brugger K Chitimia-Dobler L Dautel H Meyer-Kayser E Kahl O. Atlas of ticks (Acari: Argasidae, Ixodidae) in Germany. Exp Appl Acarol. (2021) 84:183–214. 10.1007/s10493-021-00619-1 33939100 PMC8102463

[48] Rose Vineer H. Dermacentor distribution in Germany: Maxent model supporting data. Open Sci Framework. (2022). 10.17605/OSF.IO/XK5BS

[49] Lobo JM Jiménez-Valverde A Real R. AUC: a misleading measure of the performance of predictive distribution models. Glob Ecol Biogeogr. (2008) 17:145–51. 10.1111/j.1466-8238.2007.00358.x

[50] Laaksonen M Klemola T Feuth E Sormunen JJ Puisto A Makela S. Tick-borne pathogens in Finland: comparison of *Ixodes ricinus* and *I. persulcatus* in sympatric and parapatric areas. Parasit Vectors. (2018) 11:556. 10.1186/s13071-018-3131-y 30355331 PMC6201636

[51] Eisen L Eisen RJ. Benefits and drawbacks of citizen science to complement traditional data gathering approaches for medically important hard ticks (Acari: Ixodidae) in the United States. J Med Entomol. (2020) 58:1–9. 10.1093/jme/tjaa165 32772108 PMC8056287

[52] Walter M Brugger K Rubel F. The ecological niche of *Dermacentor marginatus* in Germany. Parasitol Res. (2016) 115:2165–74. 10.1007/s00436-016-4958-9 26993325 PMC4863919

[53] Hofmeester TR van der Lei P-B Docters van Leeuwen A Sprong H van Wieren SE. New foci of *Haemaphysalis punctata* and *Dermacentor reticulatus* in the Netherlands. Ticks Tick Borne Dis. (2016) 7:367–70. 10.1016/j.ttbdis.2015.12.009 26726807

[54] Răileanu C Tauchmann O Silaghi C. Sympatric occurrence of *Ixodes ricinus* with *Dermacentor reticulatus* and *Haemaphysalis concinna* and the associated tick-borne pathogens near the German Baltic coast. Parasit Vectors. (2022) 15:65. 10.1186/s13071-022-05173-2 35193661 PMC8862291

[55] Klitgaard K Chriél M Isbrand A Jensen TK Bødker R. Identification of *Dermacentor reticulatus* ticks carrying *Rickettsia raoultii* on migrating jackal, Denmark. Ermerg Infect Dis. (2017) 23:2072–4. 10.3201/eid2312.170919 29148376 PMC5708226

[56] Alkishe A Cobos ME Osorio-Olvera L Peterson AT. Ecological niche and potential geographic distributions of *Dermacentor marginatus* and *Dermacentor reticulatus* (Acari: Ixodidae) under current and future climate conditions. Web Ecol. (2022) 22:33–45. 10.5194/we-22-33-2022

[57] Hornok S. Allochronic seasonal peak activities of *Dermacentor* and *Haemaphysalis* spp. under continental climate in Hungary. Vet Parasitol. (2009) 163:366–9. 10.1016/j.vetpar.2009.03.048 19410373

[58] Bartosik K Wisniowski L Buczek A. Abundance and seasonal activity of adult *Dermacentor reticulatus* (Acari: Amblyommidae) in eastern Poland in relation to meteorological conditions and the photoperiod. Ann Agric Environ Med. (2011) 18:340–4. 22216810

[59] German Meteorological Service [Deutscher Wetterdienst]. Monatlicher Klimastatus Deutschland - Rückblick und Vorschau. (2021). Available online at: https://www.dwd.de/DE/leistungen/pbfb_verlag_monat_klimastatus/monat_klimastatus.html (accessed October 8, 2021).

[60] Robert-Koch-Institut. FSME: Risikogebiete in Deutschland (Stand: Januar 2022). Epidemiol Bull. (2022) 2022:3–21.

[61] Chitimia-Dobler L Lemhöfer G Król N Bestehorn M Dobler G Pfeffer M. Repeated isolation of tick-borne encephalitis virus from adult *Dermacentor reticulatus* ticks in an endemic area in Germany. Parasit Vectors. (2019) 12:90–90. 10.1186/s13071-019-3346-6 30867015 PMC6416925

[62] Topp A-K Springer A Dobler G Bestehorn-Willmann M Monazahian M Strube C. New and confirmed foci of tick-borne encephalitis virus (TBEV) in northern Germany determined by TBEV detection in ticks. Pathogens. (2022) 11:126. 10.3390/pathogens11020126 35215070 PMC8876329

[63] European Centre for Disease Prevention and Control. Country Profile: Czech Republic. Tick-Borne Encephalitis (TBE). (2012). Available online at: https://www.ecdc.europa.eu/en/publications-data/country-profile-czech-republic-tick-borne-encephalitis-tbe (accessed March 21, 2022).

